# Speech Acoustic Markers Can Detect Mild Cognitive Impairment in Parkinson’s Disease

**DOI:** 10.1109/jstsp.2025.3620716

**Published:** 2025-10-17

**Authors:** Kara M. Smith, James R. Williamson, Thomas F. Quatieri

**Affiliations:** Department of Neurology, Boston Medical Center, Boston, MA 02118 USA; Massachusetts Institute of Technology Lincoln Laboratories, Lexington, MA 02421 USA; Massachusetts Institute of Technology Lincoln Laboratories, Lexington, MA 02421 USA, and also with the Speech and Hearing Bioscience and Technology Program, Harvard Medical School, Cambridge, MA 02115 USA

**Keywords:** Acoustics, cognitive dysfunction, Parkinson disease, speech

## Abstract

**Background:**

Speech biomarkers have been used to assess motor dysfunction in people with Parkinson’s disease (PD), but speech biomarkers for mild cognitive impairment in PD (PD-MCI) have not been well studied.

**Objective:**

To identify speech acoustic features associated with PD-MCI and evaluate the performance of a model to discriminate PD-MCI from participants with normal cognitive status (PD-NC).

**Methods:**

We analyzed speech samples from 42 participants with PD, diagnosed as either PD-MCI or PD-NC using the Movement disorders Society Task Force Tier II criteria as a gold-standard classification of MCI. A reading passage and a picture description task were analyzed for acoustic features, which were used to generate individual and then a final fused Gaussian mixture model (GMM) to discriminate PD-MCI and PD-NC participants.

**Results:**

The picture description task yielded a larger number of acoustic features that were highly associated with PD-MCI status compared to the reading task. Fusing the model outputs from the picture description task resulted in an AUC = 0.82 for discriminating PD-MCI from PD-NC participants. The acoustic features associated with PD-MCI stemmed from multiple speech subsystems.

**Conclusion:**

PD-MCI has a distinct speech acoustic signature that may be harnessed to develop better tools to detect and monitor this complication.

## I. Introduction

Parkinson’S disease (PD) is the second most common and fastest growing neurodegenerative disorder [1]. People living with PD (PwPD) experience motor symptoms such as bradykinesia and tremor, and well as non-motor symptoms such as depression, anxiety, and cognitive impairment [2]. Cognitive impairment in PD can range from subjective cognitive complaint to mild cognitive impairment (PD-MCI) to PD-related dementia (PDD) [3]. Speech dysfunction is another prevalent and impactful symptom that can begin early in the disease course and worsen with disease progression [4]. Speech and communication impairments commonly co-occur along with motor and cognitive decline over time. For these reasons, speech has been increasingly explored as a potential source of digital biomarkers of various clinical outcomes in PwPD [5].

Speech symptoms in PwPD are particularly complex to investigate, as motor and cognitive deficits frequently co-exist. Motor impairments typically manifest as hypokinetic dysarthria, with harsh breathy voice quality, decreased volume and prosody, imprecise articulation, and impaired speech rhythm [6]. These changes have been measured in the speech signal waveform of PwPD, and speech acoustics have been consistently characterized with markers of multiple speech subsystems including the phonatory, articulatory and respiratory subsystems. Speech acoustic measures have been increasingly supported as correlates of key underlying physiology driven by the neurobiological dysfunction in PD, including dopaminergic depletion and neural changes in basal ganglia circuits [7], [8]. Common acoustic features found in PwPD compared to controls include decreased fundamental frequency variability, increased jitter and shimmer, decreased harmonic-to-noise ratio, and decreased vowel space [5]. Speech envelope, which correlates with speech volume and forcefulness, is typically decreased in PwPD compared to controls [5]. Cepstral peak prominence (CPP), a laryngeal measure that correlates with voice quality, is decreased in early PD and may be useful in predicting disease onset [9]. Mel-frequency cepstral coefficients (MFCCs) are speech representations based on temporal trajectories of energy in different frequency bands that mimic the human cochlear frontend. MFCCs reflect how the vocal tract articulators change the speech spectrum to create different sounds and has proven to be a robust acoustic feature useful to differentiate between PwPD and controls despite different languages and recording conditions [10]. Notably, most studies using speech as a digital biomarker in PwPD have focused on discrimination of PwPD from healthy controls, and have selected participant cohorts based solely on PD-related *motor* symptoms. Thus, the speech-based algorithms currently being applied for PD are mainly informative regarding motor system integrity and cannot be used to evaluate non-motor symptoms.

Non-motor symptom monitoring using speech biomarkers would be an important advance for clinical care and research in PD, however non-motor domains, particularly mild cognitive impairment (MCI), have been relatively overlooked. In the general population, speech markers using both linguistic and acoustic features have shown success in detecting MCI [11]. Though prior research is limited, a few recent studies have reported that speech acoustic features are associated with cognitive impairment in PwPD. Articulatory acoustic measures, while previously thought to relate to speech motor processes only, have been shown to discriminate subpopulations of PwPD with poorer cognitive status [12], [13]. It has been hypothesized that cognitively demanding speech tasks may bring to light cognitively based speech dysfunction due to shared pathophysiological circuits and competing neural resources that are compromised by the frontostriatal hypodopaminergic state in PwPD [14], [15].

Lingustic deficits have been identified in PwPD during spontaneous discourse speech tasks even in early stages of neurodegeneration. Newly diagnosed PwPD with MCI displayed poorer lexical diversity and increased phrase repetitions [16]. Several studies have also demonstrated increased pausing in individuals with PD-MCI [17], [18], [19], which worsened in those with PDD [20]. While this work supports the utility of speech as a rich data source for the further development of speech biomarkers of PD-MCI, most of these studies examine only a select few speech features.

Additional research is needed to evaluate both speech acoustic and linguistic measures to develop robust, validated speech markers of cognitive impairment in PD. This will also require using rigorous criteria to define cognitive status including PD-MCI, so that there is a gold-standard ground truth underlying model development. Prior studies have used the MoCA as a proxy of global cognitive function. The MoCA was developed as a screening assessment and does not have optimal psychometric properties to accurately categorize participants for the purpose of biomarker validation [21]. On the other hand, the MDS Task Force Tier II criteria is a validated and widely recommended method for diagnosis of PD-MCI [22], but to our knowledge these criteria have never been used in speech marker development. We present the development of a speech acoustic model for discrimination of PD-MCI using the MDS Task Force Tier II criteria as a gold-standard ground truth.

Methodological factors involved in speech analysis and algorithm development should also be carefully planned for the evaluation of cognitive symptoms in PwPD. First, cognitively demanding tasks would be most appropriate to assess the multi-faceted speech and communication dysfunction in PwPD, but most studies use only reading and repetition tasks that lack substantial cognitive demand. In this work, we utilize a picture description task in which speech content varies between participants. This semi-structured speech task is more cognitively demanding than a reading passage, and will enable identification of key speech features associated with PD-MCI which can then be validated on even more ecologically relevant tasks. Additionally, methodology relating to speech signal processing may not be optimized for detection of cognitive symptom in PwPD. For instance, the handling of silent intervals varies across the literature, but may be an important processing decision point. Silent intervals (unvoiced segments and pauses) have been shown to differ in PwPD compared to controls and may be associated with cognitive decline in other patient populations [20], [23]. However, removing silent intervals is a common step taken in acoustic processing to remove noninformative parts of the audio signal and reduce noise in speech samples. This may lead to a loss of key information related to temporal dynamics of speech, but this has not been rigorously addressed in prior studies. It is unknown if the removal of silent intervals may hinder the development of speech markers for PD non-motor symptoms such as PD-MCI. In fact, there may be different optimal processing pipelines for speech data from PwPD depending on the symptom domain of interest. There is additional inconsistency across the literature in acoustic measurement settings such as time window length. Processing variations in time window lengths could impair reproducibility between studies. Thus, we explored the impact of varying time window lengths on select acoustic features in our PD-MCI model as well, in order to evaluate the robustness of the association between the features and the condition of interest.

Our objective was to identify speech acoustic markers that discriminate participants with PD-MCI from those with normal cognition (PD-NC). We conducted a cross-sectional analysis in a cohort of participants with PD-MCI and PD-NC. Participants performed two commonly applied speech tasks: a reading passage and picture description. Our feature extraction includes summary statistics that represent temporally global distributions of each acoustic feature channel, as well as summary statistics, based on auto- and cross-correlations of the acoustic feature channels, that take into account local temporal dynamics. We evaluated the efficacy of a picture description task versus a reading task in classifying PD-MCI based on changes in acoustic features across multiple speech subsystems. We also evaluated the interaction of time window length and removal of silent intervals with the type of summary statistics – global feature distributions versus local temporal dynamics – used to classify PD-MCI.

A speech assessment tool to detect and monitor cognitive impairment in PwPD has the advantages of being non-invasive, inexpensive and easily scalable to administer remotely to large patient populations. This work will help to advance speech marker research and clinical implementation in PwPD in order to improve evaluation of cognitive impairment in this disease.

## II. Methods

### A. Study Population

In a cross-sectional study, 42 participants with PD-MCI or PD-NC were initially enrolled at the University of Massachusetts Chan Medical School from 2020–2022 (Table I). All participants were diagnosed with idiopathic PD by a fellowship-trained movement disorders specialist. All participants were older than 18 years, spoke English as their primary language, and PD symptom duration was greater or equal to 2 years. Participants were excluded if they had another neurological disorder, had deep brain stimulation, or had other voice, speech, or swallowing disorders. All participants completed an informed consent procedure in accordance with the Declaration of Helsinki and approved by the institutional review board of the University of Massachusetts Chan Medical School.

### B. Assessments and Protocol

#### Clinical and cognitive assessments:

Each participant provided demographic and clinical history and underwent clinical assessments including Movement Disorders Society – Unified PD Rating Scale (MDS-UPDRS). We administered a battery of neuropsychological tests to diagnose PD-MCI by MDS Task Force Level II criteria. Clinical Dementia Rating Scale (CDRS Sum of boxes) was used to ensure that dementia was not present [20]. The Penn Parkinson Daily Activities Questionnaire (PDAQ) was administered to assess for the impact of cognitive symptoms on instrumental daily activities of living. Ref. [24] Assessments were discussed in a consensus conference attended by two movement disorders specialists and one neuropsychologist. One participant was excluded from all further analysis due to an indeterminate ruling from the consensus conference and 42 participants were included in the final analyses (Table I).

#### Speech tasks:

For the reading task, participants were asked to read the first paragraph of the Rainbow passage. For the picture description task, participants were shown the cookie theft image from the Boston Diagnostic Aphasia Examination and were asked to describe the picture to the best of their ability for 60 seconds [25].

Recording was performed using a digital recorder with a head-mounted microphone approximately 7 cm from mouth at a 45-degree angle (Zoom H4n Pro Handy Recorder and Shure WH20 headset) at sampling rate of 44100 Hz and 16 bits. The recording environment included quiet examination rooms in a clinical research unit.

### C. Feature Extraction and Acoustic Analysis

#### Acoustic features:

Acoustic time series features were computed in local processing frames. We pre-selected features a priori that have been broadly studied in PwPD [5], [10] and have been interpreted with clinical relevance [26] (Table II). These were: 1) the first three formant frequencies, generated using KARMA [27]; 2) twelve Mel-frequency cepstral coefficients (MFCCs) generated from PRAAT [28]; cepstral peak prominence (CPP), computed using AWAN [29]; and acoustic envelope (Env). For these features, we also computed feature velocities based on first-order time derivatives, denoted as dMFCC, dFormant, dEnv, and dCPP. These delta features capture the dynamic speech changes between time frames within a speech sample. Feature velocities have been shown to be useful in automated speech recognition work and to relatively robust to environmental conditions like noisy backgrounds [30]. We also calculated voice activity detections (VAD) as part of the KARMA formant tracking algorithm [27]. Intervals between voiced and unvoiced or silent segments were used to obtain information about the timing and duration of pauses during speech, including number of pauses, total duration of pauses, and pause/speech ratio.

### D. Summary Statistics of Acoustic Features

#### Temporal dynamics:

These summary statistics characterize the short-time temporal dynamics of acoustic features, both within and across the acoustic feature variables. This approach uses time delay embedding (TDE) to expand the dimensionality of time series data, providing for the representation of short-time correlations in a TDE correlation matrix. This is done at multiple delay scales to represent these correlations at multiple time frequencies [31], [32].

Given an acoustic time series with dimension *M*, the TDE embedding expands the dimensionality by the number of time delays, *N*, into an expanded time series with dimensionality *M*×*N*. The additional dimensions consist of copies of the original time series signals at *N* different time delays, with time delay spacings defined by the delay scale *S*_*d*_. A TDE correlation matrix with these embedded time delays represents not just the same-time correlation between the *M* signals, but also the correlations across multiple time delays between and within the *M* signals. The summary statistics produced by this approach consist of the TDE correlation matrix eigenvalues, concatenated across all of the time delay scales, which represent the multiscale TDE dimensionality of the signal.

A standard parameterization was used in this paper, with 5 delay scales (*d* = *1, …, 5*) and 15 time delays per scale [31]. The delay spacings for the 5 scales were *S*_*d*_ = {13,715,31} for all acoustic features. These spacings are in units of feature samples, and thus correspond to time delays of {10, 30, 70, 150, 310} ms for features sampled at 10 ms (Formants, Env, CPP), and to time delays of {5, 15, 35, 75, 155} ms for features sampled at 5 ms (MFCC).

This summary statistic approach is premised on the hypothesis that speech motor disorders such as PD involve a reduction in the complexity and coordination of articulator movements during speech [33]. This dysfunction would lead to time series dynamics with reduced intrinsic dimensionality that can be “explained” by a smaller number of eigenvectors, and thus resulting in a more skewed distribution of eigenvalues (increases in the larger eigenvalues and decreases in smaller eigenvalues). One potential strength of this approach is that the summary statistics are robust to cross-session variation in amplitude levels, which can be impacted by environmental or recording conditions.

#### Univariate distributions:

While the temporal dynamics statistics are a rich source of information about short-time dynamics and intrinsic dimensionality of acoustic features, they are blind to the absolute magnitudes and channel-dependent properties of acoustic features. In other words, the values of each acoustic variable could be randomly rescaled and (for multivariate acoustic features) the identities of the acoustic variables could be randomly shuffled without affecting the temporal dynamics statistics, which characterize only the time-frequency dimensionality of multivariate time series as a whole.

Therefore, alternative summary statistics that quantify the distribution of raw values associated with each specific acoustic variable provide useful complementary information to temporal dynamics statistics. We refer to these as univariate distribution statistics because they characterize the distribution of values of each acoustic variable across the entire recording duration. This is done using seven standard statistical measures: mean, standard deviation, skewness, kurtosis, median, maximum, and minimum. It has previously been found that fusion of temporal dynamics and univariate distribution statistics improved speech-based estimates of the deleterious effects of sleep deprivation on cognitive performance [32].

#### Pauses:

A final set of summary statistics characterize the timing and durations of pauses. The pause statistics are: the number of pauses, the average pause duration, and the total duration of the speech utterance, which provides context for the number of pauses.

Table III lists the summary statistics, which are computed across an entire speech recording. The temporal dynamics and univariate distribution statistics are computed for each of the 8 low-level acoustic time series features: MFCC, dMFCC, Formant, dFormant, Env, dEnv, CPP, and dCPP. The dimensionality of the summary statistics depends on the acoustic feature dimensionality, M. These summary statistics are then used as features in classification and regression models, as described below.

### E. Impact of Variation in Window Length and Handling of Unvoiced Segments on Acoustic Features

Differences in acoustic feature processing could have an impact on the values obtained in automated processing pipelines, thus diminishing reproducibility. Because the temporal dynamic and univariate distribution summary statistics quantify different qualities of the speech signals, it is possible that acoustic feature processing details could have different impacts on each type of summary statistic. One potential source of differential impacts concerns the handling of short non-speech silences, including unvoiced segments and silent pauses. For temporal dynamics statistics, it has previously been found that including short duration silences produce a better characterization of the full dynamics [30], [31]. However, for the univariate distribution statistics, it may be preferrable to focus exclusively on the speech signal, ignoring noisy acoustic features during non-speech silences [34]. We therefore evaluated whether removal of unvoiced segments altered the acoustic features or the final model for PD-MCI. We accomplished this by applying the VAD from the KARMA formant tracking algorithm to the MFCC features and assessing how the inclusion of features with and without VAD impacted the discrimination of PD-MCI in the model.

We next assessed whether varying MFCC time windows would have a different impact on the temporal dynamic features versus the univariate distribution features in the PD-MCI model. Due to the tradeoff between temporal and frequency resolution involved in choice of window size [35], it is possible that summary statistics quantifying temporal dynamics might be optimized at a different window size than those quantifying global feature distributions. The time windows used for computing MFCCs were 5, 10, 15, and 20 ms. The findings from both the VAD and time window investigations were then used to determine the appropriate acoustic feature processing for computing the summary statistics in all subsequent analyses.

### F. Machine Learning Model Development

#### Classification and regression models:

For each low-level feature, temporal dynamics and univariate distribution summary statistics were computed and input separately into a Gaussian mixture model (GMM) classifier to discriminate our primary endpoint, PD-MCI or PD-NC. For each low-level acoustic feature and high-level summary statistic, a background GMM was formed from the pooled data across all training subjects. The background GMM was then adapted using the technique in Reynolds et al. [36]. The output of the GMM is the log-likelihood ratio between the PD-MCI GMM and the PD-NC GMM. A similar approach that used an ensemble of adapted GMMs was used for tracking depression severity from speech in [31].

#### Cross-validation and feature selection:

Detection accuracy with leave one subject out (LOSO) cross-validation was quantified using the area under the ROC curve statistic (AUC) computed across all subjects. Nested LOSO cross-validation was also used to automatically select the PCA dimensionality from each training set fold to be applied to the test fold. Within the nested cross-validation, the best performing number of principle components (PCs) was selected, from a range of 1 to 15 PCs. In addition, when fusing multiple GMMs (each combination of acoustic feature and summary statistics produces a different GMM), the AUC obtained in the nested cross-validation was used for feature selection, based on a default AUC threshold of 0.75. The feature selection AUC threshold was also systematically varied to evaluate sensitivity of fused classification performance to this parameter. Fusion involved averaging the GMM outputs (log-likelihood ratios) of all suprathreshold GMM outputs. If, for a given training fold, the validation AUCs for all GMMs were subthreshold, then the single model with the highest validation AUC was used.

As a secondary aim, we evaluated associations between the previously described acoustic features and PD motor severity. We did this in order to understand how our speech analysis pipeline designed for detection of PD-MCI may work for the more frequently studied outcome of PD motor symptoms. Using the MDS-UPDRS Part III motor score as the dependent variable, we performed a cross-validation procedure with a multivariate linear regression model. Here, the number of PCs was selected using the Spearman correlation in the validation set as the selection criterion.

### G. Effect Size Analysis

We computed and plotted the Cohen’s d effect sizes for PD-MCI on the picture description task. This was done for the mean and standard deviation summary statistics for MFCCs, Formants, Env, and CPP, as well as the Pause summary statistics. This was done to enhance interpretability and clinical relevance, by providing insight into the nature of detectable speech changes that are associated with PD-MCI.

## III. Results

### A. Acoustic Feature Parameter Analysis: Variation in Window Length and Handling of Unvoiced Segments

**Fig. 1.**
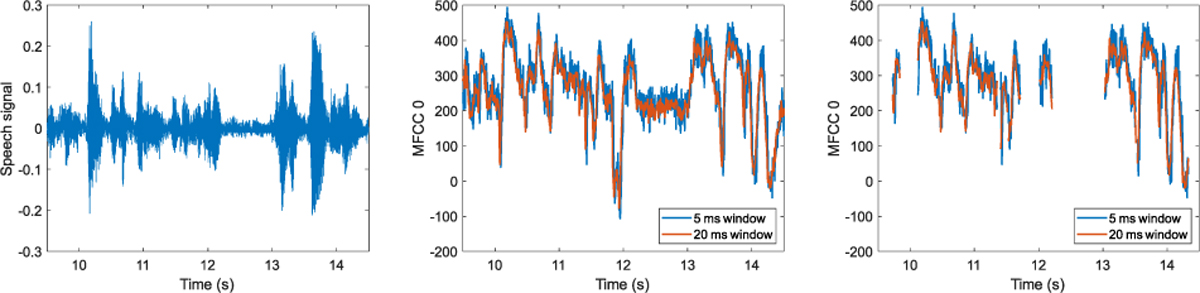
Analysis variations for MFCC coefficients. Left: 5 s speech segment from picture description task: “ …probably end up getting the cookies too …mother drying dishes.” Middle: MFCC0 coefficients computed from sliding 5 ms and 20 ms processing windows. Right: MFCC0 coefficients at 5 ms and 20 ms resolution after removal of unvoiced segments.

Altering the time windows affects the MFCC features, as is demonstrated on a speech example extract from the picture description task in Fig. 1. Increasing the time window increases the range of data used to estimate the MFCC coefficients, reducing the noise (Fig. 1, middle). Removal of unvoiced segments introduces discontinuities in local time trajectories of the MFCC coefficients. When incorporated into a machine learning model to detect PD-MCI, these MFCC processing variations can affect model performance, depending on the type of summary statistics used to characterize MFCC feature time series, as is shown in Table IV. Using the temporal dynamics statistics of MFCC features, inclusion of unvoiced segments (i.e., no VAD used) produces a higher AUC. In addition, accuracy monotonically improves as the MFCC time window is decreased, and the highest accuracy (AUC = 0.84) is obtained witha5 ms window. Univariate distribution statistics, on the other hand, produce better results with removal of unvoiced segments (i.e., VAD used), and the highest accuracy (AUC = 0.87) is obtained with a 20 ms window.

Due to the results of this exploratory analysis, all subsequent analysis was done with the following parameter settings. For temporal dynamics statistics, unvoiced segments were not removed for any acoustic features, and a 5 ms window was used to compute MFCCs. For univariate distribution statistics, VAD was used to remove unvoiced segments for all acoustic features, and a 20 ms window was used to compute MFCCs.

### B. Speech Acoustic Measures Associated With PD-MCI

**Fig. 2.**
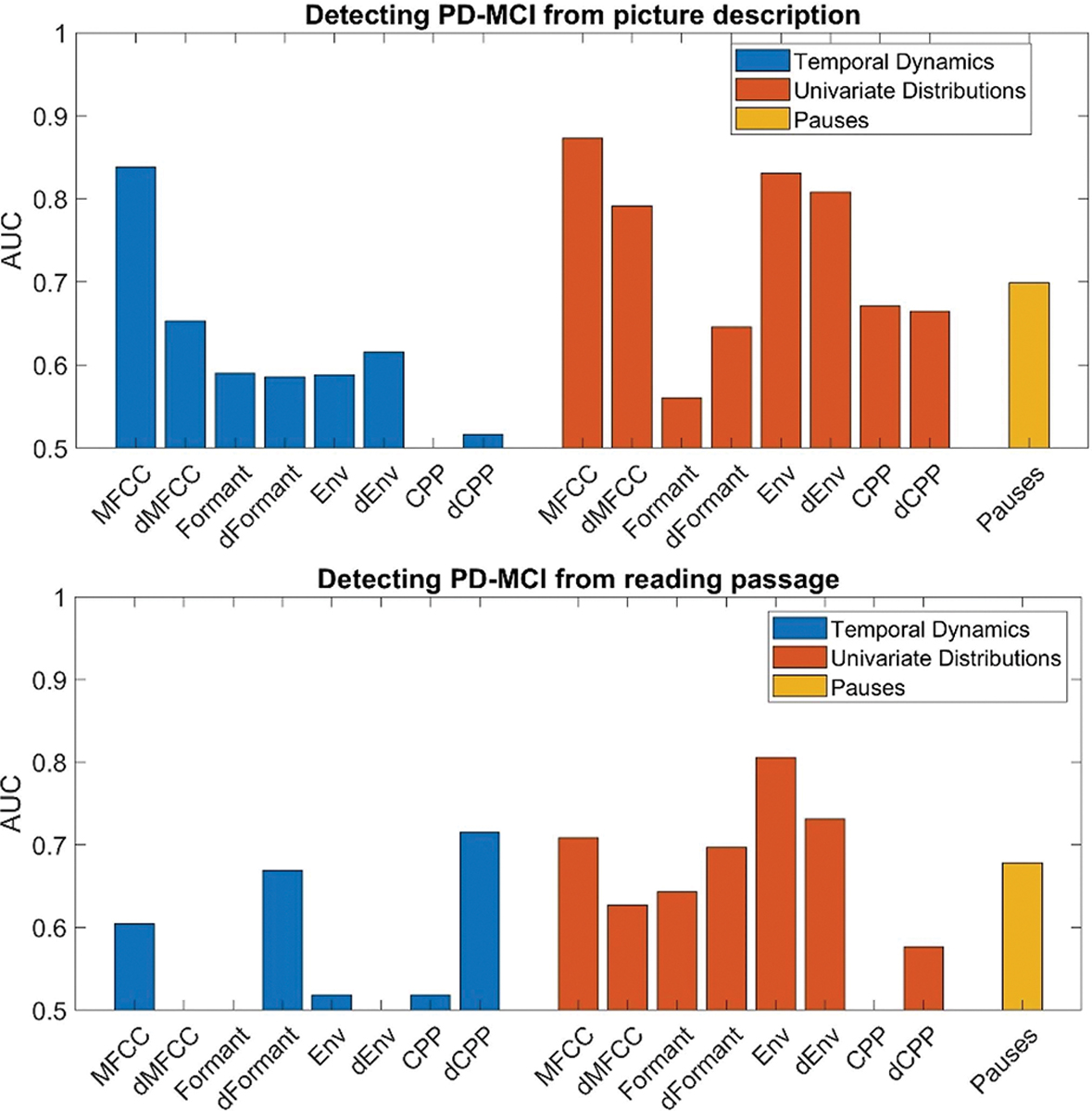
Average AUC values in discriminating PD-MCI from PD-NC. The average AUC values obtained from using summary statistics of different acoustic features are shown, based on the picture description (left) and reading passage (right).

Our primary aim was to discriminate PD-MCI from PD-NC. Fig. 2 plots AUCs obtained in classifying PD-MCI using temporal dynamics, univariate distribution, and pause summary statistics from the picture description task (top) and reading task (bottom). Temporal dynamics of MFCCs produced a high AUC (AUC = 0.84) based on picture description. However, there are no other acoustic features that produce strong AUCs using temporal dynamics for either task. Univariate distributions produced high AUCs using MFCCs and delta-MFCCS (AUC = 0.87 and 0.79) and Envelope and delta-Envelope (AUC = 0.83, 0.81) on picture description. The pause features also performed moderately well, with AUC = 0.70. The reading passage task yielded a smaller number of predictive features: delta-CPP produced an AUC of 0.72 using temporal dynamics statistics, while MFCCs produced an AUC of 0.71, and Env and delta-Env produced AUCs of 0.81 and 0.73 using univariate distributions.

### C. Speech Acoustic Measures Associated With Motor Severity

**Fig. 3.**
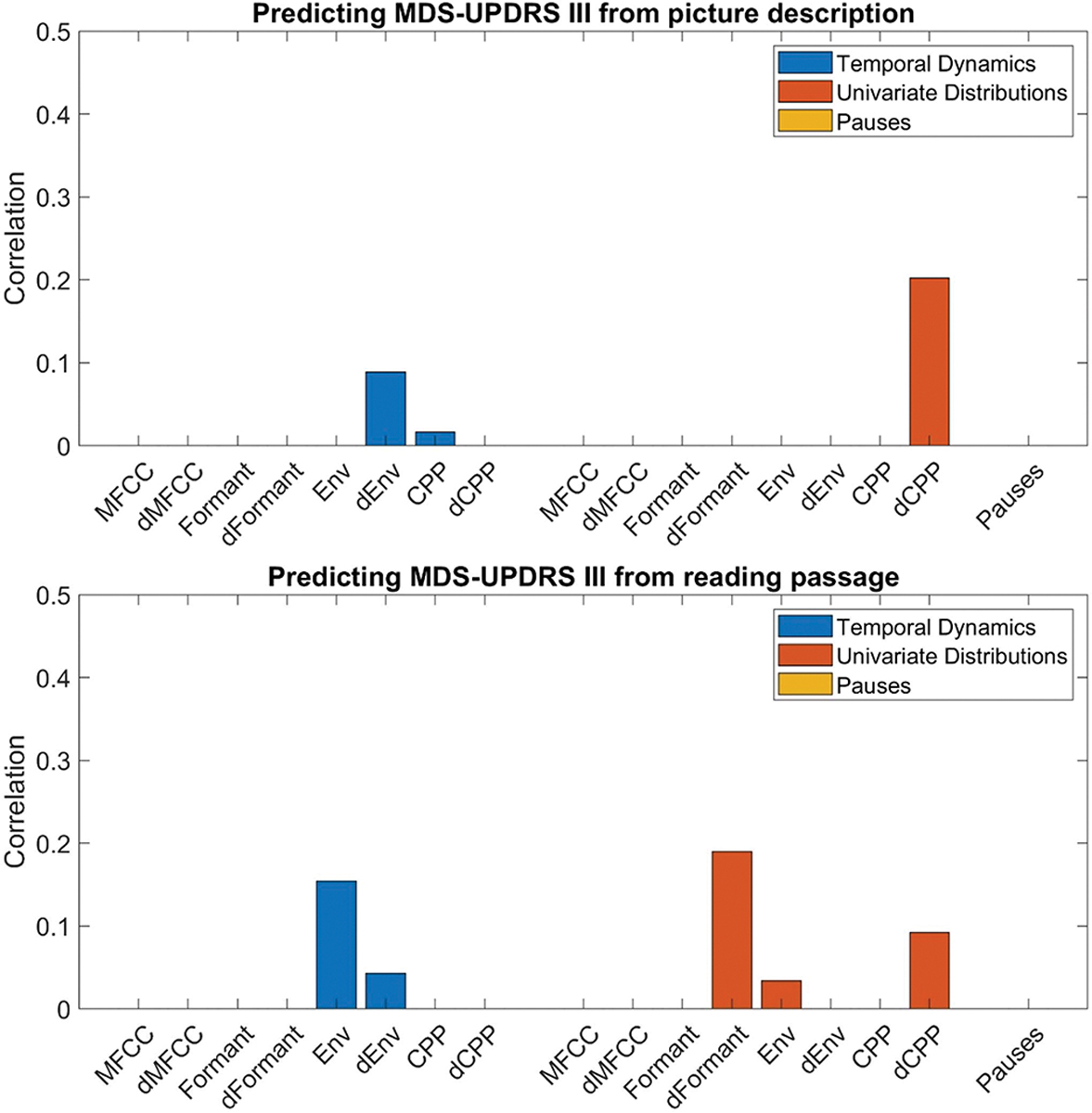
Average Spearman correlations in predicting motor symptom severity. The Spearman correlation coefficients (average R) from linear regression are presented, using the motor symptom severity score (MDS-UPDRS III) as the dependent outcome and the summary statistics of different acoustic features as independent variables, based on the picture description (left) and reading passage (right).

As a secondary exploration, speech acoustic features used in the PD-MCI model were then assessed for their association with motor severity. Using the MDS-UPDRS Part III motor score (UPDRS) as the dependent variable, linear regression led to a low degree of association for all acoustic features and pause measures (Fig. 3). Spearman correlation coefficients for the linear regression were all <0.3, indicating a weak association.

### D. Fusion of Speech Acoustic Measures for Detecting PD-MCI

Fusion of multiple feature models was done using feature selection determined by validation AUCs, as described in Section II-F. Table V shows the AUCs for detecting PD-MCI that are obtained by considering fusion within and across the summary statistics for picture description and reading passage tasks using an AUC feature selection threshold during nested cross-validation of 0.75. The best result uses fusion across all features on the picture description (AUC = 0.82) with slightly lower results of AUC = 0.79 using only temporal dynamics and 0.81 using only univariate distributions. Table VI shows that the fused AUC is robust to changes in the AUC feature selection threshold, with AUC > = 0.81 provided the feature selection threshold is > = 0.70.

### E. Feature Effect Sizes for PD-MCI

**Fig. 4.**
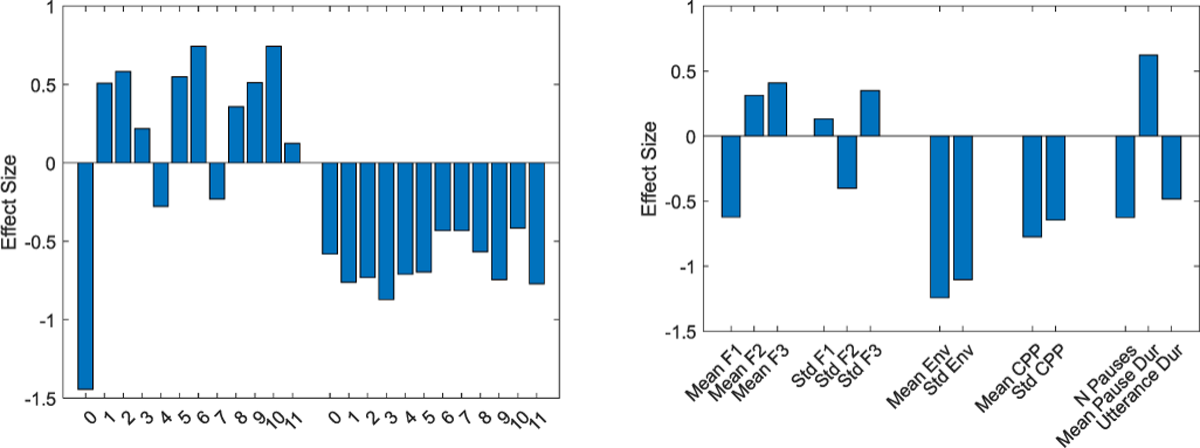
Effect sizes for PD-MCI relative to PD-NC for univariate distributions. Cohen’s D values are shown for discriminating PD-MCI and PD-NC using the mean and standard deviation of MFCCs (left), and for Formants, Env, CPP and pauses (right).

By plotting the effect sizes of mean, standard deviation, and pause summary statistics for PD-MCI from the picture description task, we assessed the relative strength of the various speech changes due to PD-MCI in univariate distributions (Fig. 4). The largest effects are seen for reduction in speech energy (MFCC0) and amplitude of speech envelope (Env, dEnv) in PD-MCI. For mean MFCCs beyond speech energy, we see similar positive effect sizes of many of the coefficients, implying roughly equal importance for most spectral components. In addition, all MFCC coefficients show a negative effect size for standard deviation, indicating a reduction in variability over time in all spectral bands. The pause features show that PD-MCI is associated primarily with longer pause duration, as the reduction in average pause number coincides with a reduction in total utterance duration.

## IV. Conclusion

We identified speech acoustic measures, associated with both temporal dynamics and longer-term univariate distributions, sensitive to detecting MCI in PwPD. Overall, our findings indicate that cognitive impairment, even when in mild stages, has a substantial impact on speech acoustic measures. We propose that our findings using a picture description task and based on rigorous PD-MCI diagnostic criteria may offer a robust foundation for the ongoing development of speech markers of PD-MCI. Consistent with our hypothesis, a picture description task outperformed a simple reading task in identifying acoustic measures associated with PD-MCI status. This is likely because picture description requires cognitive input including narrative organization, lexical retrieval, and syntax planning. Our results are consistent with prior work showing that speech and language abnormalities could be detected in the early stages of PD using cognitively-demanding speech tasks [16].

Overall, we identified several acoustic features that discriminate PD-MCI and PD-NC. MFCC coefficients were effective in discriminating PD-MCI in the picture description task, with the negative effect sizes for standard deviations in all 12 coefficients indicating a general reduced variability in MFCCs over time in PD-MCI. Interestingly, for MFCC coefficients, both temporal dynamics and univariate distributions were strongly associated with PD-MCI status. We also identified a strong association between envelope features and PD-MCI, suggesting a less forceful voice is associated with PD-MCI. This association was stronger for the univariate distributions of envelope features. Envelope is linked with sound pressure level, with underlying physiology related to speech volume and forcefulness. In PwPD, either increased or decreased subglottal pressure, depending on the speech content, could result in decreased envelope [5]. A lack of coordination between vocal apparatus musculature and respiratory control may result in mismatch to the desired speech output [38]. Cognitive impairment may worsen this mismatch, as it was observed in the PD-MCI group during both reading and picture description tasks in this study. Though envelope is usually considered a motor speech measure, we did not find a strong association of envelope with motor symptom severity in our cohort. In general, the acoustic measures that performed best for discriminating PD-MCI from PD-NC demonstrated only modest association with motor symptoms. Further research is warranted to elucidate the relative motor and cognitive contributions to these features, such as envelope measures. However, our interpretation of these results is that the distinct acoustic signature of PD-MCI compared to that of PD-NC is not substantially confounded by motor deficits. Given that the motor symptom severity was not significantly different between these groups, the distinct acoustic differences we identified are more likely related to the cognitive and communication symptoms experienced by the participants.

Also consistent with prior literature, we found that pause duration was increased in PD-MCI. Both pause duration and speech acoustic measures showed a high performance in the PD-MCI model, highlighting that several speech subsystems can be queried to detect PD-MCI in addition to the cognitive-linguistic system. Though our study was not designed specifically to determine how cognitive-linguistic measures compare to motor speech-based acoustic measures in discriminating PD-MCI, our interpretation is that both types of features are useful tools that could be further developed to detect PD-MCI in the clinic. Such multi-faceted speech-based diagnostic tools may prove particularly beneficial for speakers with different primary languages or complex symptomatology. Whereas pauses may be affected by language/dialect, speech task type, fatigue and mood, the other acoustic markers of PD-MCI we identified could be added to increase the sensitivity and specificity of such tools.

No prior studies have thoroughly evaluated acoustic measures in a well-characterized PD-MCI cohort. Prior work is limited to a few small studies including MoCA to categorize global cognitive status. This work has shown that consonant imprecision and articulatory measures may be associated with a decline in global cognition over time as measured by MoCA in PwPD [39]. It is notable that we found additional speech subsystems to be associated with cognitive status, including laryngeal and phonatory subsystem markers. Garcia et al. examined 40 participants with PD with and without MCI (categorized by MoCA) and 40 controls using story reading and story retelling tasks performed in Spanish. Several acoustic measures performed well in discriminating both PD groups from controls, but the poorest model performance was in discriminating PD-MCI from PD-NC. Only a single complex phonemic identifiability feature in the retelling task was significantly associated with PD-MCI vs. PD-NC with AUC in a lower range at 0.76 [12]. We highlight that our fused model was able to discriminate PD-MCI from PD-NC with AUC = 0.82. Some of the differences between these prior studies and our findings may be explained by differences in our participant characteristics, criteria used to classify cognitive status, primary language used, and selected speech task. In summary, despite differences in the specific speech biomarkers used, our findings are consistent with the literature in demonstrating that cognitive status in PwPD is distinctly associated with speech acoustic impairments. One potential mechanism may be that when talkers with PD-MCI perform cognitively demanding tasks, their ability to maintain speech quality becomes more strained and multiple speech subsystems become impaired. Prior work has suggested that tasks that require cognitive skills like working memory [40] and executive function [41] may require utilization of compensatory mechanisms in PD-MCI because frontostriatal circuits suffer from a hypodopaminergic state as dopamine degeneration worsens in the basal ganglia [3]. Given that speech motor control also relies on basal ganglia pathways [42], the added cognitive demand of a speaking task in a talker with PD-MCI might thus lead to worsened compensatory resilience and further speech acoustic impairments. This hypothesis warrants further investigation applying advanced biomarkers and functional neuroimaging.

We also sought to understand how motor symptoms were associated with the speech markers in our model. While our primary aim was to identify speech acoustic markers of PD-MCI, it is the motor symptom domain that has dominated much of the PD speech marker literature. Thus, to be comprehensive in our exploration of our highest performing speech acoustic features, we evaluated their association with the motor severity score of the MDS-UPDRS. Features such as MFCC, CPP and envelope, are usually considered relevant to speech motor function. While we did not identify a strong relationship between motor symptom severity and our selected speech acoustic measures, there are several possible explanations for this seemingly contradictory result. First, using the MDS-UPDRS Part III total score may not be a sensitive outcome measure for the specific motor symptoms likely to impact the vocal apparatus. Using single items like the clinician-rated speech item may focus more on the vocal system, but there are statistical validity issues with using just this 4-point item in isolation [43]. Speech motor dysfunction is heterogeneous in PwPD, thus the relationship between motor symptoms, speech motor deficits and global communication impairment is also variable and requires further study. In line with our primary objective, we selected our speech tasks, feature analysis pipeline, and model development toward detecting PD-MCI rather than estimating the MDS-UPDRS score. In a future larger study, it may be explored if and how much overlap exists between speech markers for motor vs. non-motor symptoms. Based on our findings thus far, we hypothesize that distinct speech makers may be useful to classifying different clinical symptoms or domains in PwPD. While another possible approach would be to use “cognitive-linguistic” features like lexical, grammatical and pause measures to evaluate cognitive symptoms and standard motor speech features to evaluate motor symptoms, we explored both types of measures in an unbiased fashion. This combined approach may fuel novel speech-based assessment tools for PwPD. Just as PwPD experience heterogeneity in motor and non-motor symptom onset and progression, speech-based markers can become more multifaceted and personalized, leading to individualized monitoring of progression and response to therapies.

Another key component of our findings relates to interaction of summary statistic approaches to feature extraction methodologies. Our analyses showed that removal of unvoiced consonants and non-speech pauses had a negative effect on summary statistics that characterize temporal dynamics but a positive effect on statistics that characterize global feature distributions. We note, however, that removal of unvoiced consonants may also be a limitation when studying certain types of speech disorders and should be considered carefully. Regarding the effect of window size, signal processing feature analysis typically contains a trade-off between temporal and frequency resolution [35]. We explored this tradeoff in the context of MFCCs. We found that shorter window lengths allowed for improved assessment of acoustic measures that vary dynamically and that longer window lengths were preferable for acoustic measures that depend on stable measurements. Our findings contribute to the literature highlighting that commonly used acoustic features may vary based on the technical approach utilized [44]. Our results lend support to the call for harmonized protocols to enhance replicability between studies.

Importantly, by exploring how these processing variations impacted our data, we have confirmed the robustness of our PD-MCI model. It is possible that different feature extraction and processing techniques may lead to more accurate models for particular symptom domains in PwPD, such as depression, anxiety and fatigue. Different machine learning approaches may also be worthwhile to explore in future work. Notably, the innovation in this current work lies in the detection of a relatively understudied non-motor symptom in PwPD, rather than in the machine learning methodology. Other promising tools beyond GMMs could further augment future research in detecting and disentangling domains of symptoms in PwPD. [45], [46], [47].

### A. Strengths and Limitations

Strengths of our study include the use of the MDS Tier II criteria for diagnosis of PD-MCI as a ground-truth. Additionally, we selected acoustic features that have been consistently shown to be impacted by PD in a large number of studies over many years, supporting the external validity of our resultant model. Our modeling approach using nested LOSO cross validation for parameter and feature selection was performed to avoid overfitting in machine learning models.

Limitations include our sample size, which is within the typical range for this type of clinical study but which may be a limitation for machine learning model development and validation. In addition, the algorithms in the processing programs we utilized for acoustic feature extraction have been developed primarily using data from healthy individuals. Thus, the accuracy and appropriateness of these approaches, importantly the voicing detector, should be evaluated in PwPD before being widely used in future studies. Another potential limitation is the selection of window length parameters. Since we found that adjusting the window length could impact our model, we stress that further work is needed to establish widely accepted guidelines for the selection of windows and other processing parameters. Such work will allow for replicable and robust speech marker tools. In terms of the speech acoustic features that we evaluated, one limitation may be that most of these features have been studied in connection with motor speech deficits and require further research to understand the mechanisms underlying their cognitive correlates.

Additionally, we used a single pause measure which included only silent intervals and their duration relative to voiced segments. This approach did not allow us to evaluate filled pauses and other types of speech dysfluencies that are common in PD-MCI. Also noted is that use of a voicing activity detector will also impact unvoiced consonants (e.g., /sh/). This will impact certain features that we used in our models to a lesser degree, including CPP or formants. There may be more of an impact on features like MFCCs and envelope which needs to be considered in future research. We also did not account for speech rate or articulatory rate in our approach. In future work, it will be useful to study more naturalistic connected speech tasks and evaluate these detailed pausing and timing features. In terms of our sample, the relatively small number of participants is another potential limitation. The recordings occurred in a clinical research center, and while all spaces were relatively quiet, we did not control for the specific rooms used for each participant. We did not account for age or sex-related differences in our models. However, there were no significant differences in age or sex between the PD-MCI and PD-NC groups.

In conclusion, speech-based digital biomarkers hold promise for evaluating non-motor symptoms in PwPD. Speech markers may be a groundbreaking and innovative approach to more easily and equitably detect and monitor cognitive impairment from its earliest stages in PwPD.

## Figures and Tables

**TABLE I:** Demographic and Clinical Characteristics of PD Participants

	PD-MCI (n=24)	PD-NC (n=18)	
	Mean (SD)	Mean (SD)	p-value
Age (years)	67.7 (7.4)	67.5 (7.7)	0.934
Female, n (%)	9 (36%)	6 (28.6%)	0.592
Education (years)	15.5 (3.5)	17.3(2.1)	0.040
MOCA	26.4 (2.6)	28.5 (1.3)	0.001
PDAQ Total	173.9 (22.7)	169.0 (23.1)	0.472
MDS-UPDRS III	30.7 (10.0)	30.9 (12.4)	0.961
MDS-UPDRS II Speech item			0.592
No speech symptoms (0) n (%)	6 (24%)	7 (33.3%)	
Slight speech symptoms (1)	13 (52%)	10 (47.6%)	
Mild speech symptoms (2)	6 (24%)	4 (19.1%)	

PD-MCI, Paikinson’s disease with mild cognitive impairment; PD-NC, Parkinson’s disease with normal cognition; MOCA, Montreal cognitive assessment; PDAQ, Penn Parkinson’s disease Daily Activities Questionnaire; MDS-UPDRS III, Movement disorders Society-Unified PD Rating Scale Part III Motor examination total score; MDS-UPDRS 2.1 Speech item, Movement disorders Society-Unified PD Rating Scale Part II item 1 which asks participants to self-report their speech symptoms as none (0), slight (1), mild (2), moderate (3), or severe (4). SD, Standard deviation. Pairwise comparisons between the means for these clinical variables are based on t-test for continuous variables and analysis of variance (ANOVA) for binary or categorical variables.

**TABLE II:** Description of Acoustic Features Selected for Analysis

Acoustic Feature	Sampling rate	Number of variables	Description
MFCC	200 Hz	12	Short-term spectral features modeled on human auditory perception, providing information about timbre, vocal tract shape, speech phonetics, and emotional content
Formant	100 Hz	3	Resonant frequencies of the vocal tract, providing information about vowel identity, articulatory configuration, and emotional content
Env	100 Hz	1	Smooth contour of speech amplitudes that offers insights into prosodic structure, timing, and speech rhythm, which are important for both intelligibility and emotional expression.
CPP	100 Hz	1	Quantification of strength of pitch signal relative to other speech sounds, sensitive to voice quality, glottal periodicity, and degree of phonation
VAD	100 Hz	1	Voice activity detections that provide information about timing and duration of pauses

MFCC, Mel-frequency cepstral coefficients; Env, envelope; CPP, cepstral peak prominence (computed using AWAN); VAD, vowel activity detection (computed using KARMA). First-order derivatives representing feature velocities were also computed for the MFCC, Formant, Env and CPP features.

**TABLE III:** Types of Summary Statistics Computed From Acoustic Features for Each Speech Recording

Summary statistics	Dimensionality	Description
Temporal dynamics	M * 5 * 15	Concatenated eigenspectra from 5 TDE correlation matrices at different delay scales, with 15 delays per scale
Univariate distributions	M * 7	Global statistics of each acoustic variable: Mean, std, skewness, kurtosis, median, maximum, minimum
Pauses	3	Computed from VAD only. Number of pauses, average duration of pauses, and speech recording duration.

TDE, time delay embedding; VAD, voice activity detection. The dimensionality depends on the number of variables, M, of each acoustic feature.

**TABLE IV:** Result of Acoustic Feature Parameter Variations in MFCC Acoustic Feature on Ability to Detect PD-MCI Using Temporal Dynamics and Univariate Distribution Summary Statistics

MFCC summary statistics	VAD filtering	AUC detecting PD-MCI from picture description task			
		5 ms	10 ms	15 ms	20 ms
Temporal dynamics	No	0.84	0.79	0.79	0.77
	Yes	0.68	0.60	0.51	0.59
Univariate distributions	No	0.61	0.66	0.66	0.66
	Yes	0.72	0.83	0.85	0.87

MFCC, Mel-frequency cepstral coefficients; VAD, voice activity detection; PD-MCI, Parkinson’s disease with mild cognitive impairment AUC values are presented for discrimination between PD-MCI and PD-normal cognition (PD-NC).

**TABLE V:** AUC Results in Detecting PD-MCI Based on Fusion of Summary Statistics Features, Based on the Picture Description (Top) and Reading Passage (Bottom)

Speech task	Summary statistic	AUC
Picture description	Temporal dynamics	0.79
	Univariate distributions	0.81
	Pauses	0.70
	Fused	0.82
Reading passage	Temporal dynamics	0.34
	Univariate distributions	0.58
	Pauses	0.68
	Fused	0.57

**TABLE VI:** AUC Results in Detecting PD-MCI: Fused Results as a Function of Feature Selection Threshold

AUC feature selection threshold	Fused PD-MCI detection AUC
0.00	0.79
0.50	0.79
0.55	0.79
0.60	0.79
0.65	0.77
0.70	0.81
0.75	0.82
0.80	0.81
0.85	0.82

Fused AUC results for discriminating PD-MCI from PD-NC, displayed with corresponding AUC feature selection threshold which was varied to demonstrate robustness of the AUC result.
